# Effectiveness of an intervention in groups of family caregivers of dependent patients for their application in primary health centers. Study protocol

**DOI:** 10.1186/1471-2458-10-559

**Published:** 2010-09-17

**Authors:** Emiliano Rodríguez-Sánchez, Sara Mora-Simón, Nieves Porras-Santos, Maria C Patino-Alonso, José I Recio-Rodríguez, Concepción Becerro-Muñoz, Diana Pérez-Arechaederra, Manuel A Gomez-Marcos, Luis Garcia-Ortiz

**Affiliations:** 1 Primary care research unit of La Alamedilla Health Center, Castilla y León Health Service - SACYL, Salamanca, Spain; 2 Departamento de Estadística. Universidad de Salamanca. Salamanca. Spain

## Abstract

**Background:**

Although Primary Health Care (PHC) Teams are used to deal with prevention and treatment of sanitary problems in adults with chronic diseases, they usually have a lack of experience in development of psychotherapeutic interventions. However, these interventions are the ones that achieve better results to reduce symptomatology and improve emotional state of caregivers.

The study aims to evaluate the effectiveness of an intervention of psychotherapy in improving the mental health and Quality of life of caregivers. This intervention is based on theoretical approaches to care adjusted to cognitive theory, in order to be applied in primary health care centres.

**Methods/Design:**

This is multicentre clinical trials study, randomized in two parallel groups, carry out in two PHC, Study population: 150 caregivers will be included by consecutive sampling and they will be randomized the half to experimental group and the other half to control group. They provide mostly all the assistance to care-dependent familiars receiving attention in PHC Centers.

Measurements: Each caregiver will be evaluated on a personal interview. The caregivers' assessment protocol: 1) Assessment of different socio-demographic related to care, and caregiver's personal situation. 2)Care-dependent individuals will also be assessed by Barthel Index and Pfeiffer Questionnaire (SPMSQ). 3)Change in caregivers will be the principal measure: family function (Family APGAR Questionnaire), burden short questionnaire (Short Zarit Burden Interview), quality of life (Ruiz & Baca: 1993 Questionnaire), the Duke-UNK Functional Social Support Questionnaire, the General Health Questionnaire-12, and changes in Dysfunctional Thoughts about caring. 4) Intervention implementation measures will also be assessed.

Intervention: A psychotherapeutic intervention will be 8 sessions of 90 minutes in groups. This intervention has been initially developed for family caregivers of patients with dementia.

**Discussion:**

Psychotherapeutic interventions have been proved to obtain better results to reduce symptomatology and improve emotional state of caregivers. Moreover, this intervention has been proved to be effective in a different setting other than PHC, and was developed by professionals of Mental Health. If we found that this intervention is effective in PHC and with our professionals, it would be an important instrument to offer to caregivers of care-dependent patients.

**Trial Registration:**

ClinicalTrials.gov Identifier NCT01177696

## Background

Nowadays, family caregivers are one of the most important resources in the care provided to dependent patients. In numerous occasions the responsibility they face causes physical, mental and economic alterations that might lead to the resignation of the family caregiver, as well as to the deterioration of the quality of life and the institutionalization of the patient under care [1-3].

The great number of occurrences of mental health disorders caused by the care given to the dependent population is corroborated by several studies affirming that the restriction of social activities, insomnia, psychological discomfort, despair, excess, stress, physical problems, difficulties in the work and professional sphere, emotional disruption (anxiety, depression), reduction of leisure time [4] and feelings of discomfort regarding life in general, are just some examples that indirectly deteriorate the quality of life of the caregiver, the patient and the family unit [5,6]. An appropriate service to the caregiver, working alongside the social services and establishing measures to combine family and professional life may contribute to prevent or palliate the so-called "ill caregiver syndrome", increase his or her self-esteem and quality of life, improve the care to a dependent person and avoid his or her institutionalization [3]. Several different educational programs have been developed with data indicating a significant increase of the caregiver's knowledge on the development of the relative's disease and on his or her competence. However, their effectiveness to reduce the caregiver's discomfort has not been proven yet [7]. In fact, in some cases this type of programs causes tension, rather than reducing it, but usually include information about problems that do not affect the patient under care, anticipating future problematic situations that might never occur. Moreover, it has been verified that a better knowledge about the disease of the dependent patient does not correlate with a lesser emotional discomfort on the part of the caregiver [8,9]. This reveals the necessity to evaluate the effectiveness of non-pharmacological therapies.

Psychotherapeutic interventions achieve best results in reducing the symptomatology and improving the emotional state of the caregivers [7]. However, in order to develop those intervention programs, which alleviate the burden of the caregiver, they have to be designed from a multi-dimensional and multi-professional perspective [10]. In addition, the intervention must be carried out by well-trained professionals with the capacity to face, control and solve their emotional problems.

For a long time, Primary Health Care Teams have been dealing with the prevention and treatment of health problems in adults with chronic diseases. There are the professionals who offer health care to dependent patients as well as to their caregivers. Although their necessities are covered by the Primary Health System, it is an enormously difficult task, mainly because health care professionals lack the appropriate training to perform basic duties needed in this field. According to a study carried out in Primary Health Care System [11], three out of 4 health care professionals feel competent to attend demands related with routine care tasks; just 5.5 out of 10 feel competent in advising on problematic behavior and problematic situations related to health care, whereas only 3 out of 10 feel qualified to offer some guidance on the consequences of such care. A vast majority of professionals believe that there is a need to train the caregivers on psychosocial and behavioral aspects so as to more accurately assist them on their necessities. Given into account that 7 out of 10 claim not to have received any kind of training on this matter, it seems logic to assume that the majority of the necessities put forward directly or indirectly by the caregivers are at present not been dealt with. Thus, it is essential to invest on professional training on a field with a great impact on the personal, social and political levels.

By means of a standard psychotherapeutic intervention, Losada et al.[12] achieved to modify a variety of problematic behaviors in groups of family caregivers of patients suffering from dementia.

This essay attempts to analyze a psychotherapeutic intervention from a comprehensive perspective similar to that of Losada with caregivers of patients suffering from different pathologies in the primary health care context.

The following objectives have been established:

1.- Designing and assessing a psychotherapeutic intervention strategy in the Primary Health Care System to efficiently work on the mental health and the quality of life of the family caregivers of dependent patients.

2.- Assessing a cognitive-behavioral intervention, which is to be applied on Primary Health Care Centers, in order to modify the dysfunctional beliefs so as to appropriately deal with the care given, based on the principles of the cognitive theory.

## Methods/Design

### Design

This is a multicentered, randomized, controlled, clinical trial to assess the effectiveness of a group intervention on the family caregivers of dependent patients.

### Study population

The study will be carried out at the Primary Health Care System in urban areas. The study subjects will be adults both male and female living near two health centers in the city of Salamanca (La Alamedilla, Miguel Armijo), an estimated population of 42.000 residents. The health care professionals of both centers will offer the caregivers of dependent relatives the opportunity to participate on the study. Once the informed consent is signed, a researcher will complete the preintervention assessment by means of an individual interview.

The sample size is expected to reveal a difference of or superior to 12 units on the Ruiz-Baca's quality of life questionnaires between the intervention group and its control. With an estimated alpha risk 0.05 and a beta risk 0.20 in a bilateral contrast, 73 subjects are required in the first group and 73 in the second one. The common standard deviation assumed is 25.7 from a previous study.

Through consecutive sampling, 150 caregivers will be included, who are responsible for almost all the care given to dependent and ill relatives who receive health care in the centers. One half will be randomly assigned to the trial group and the other half to the control group in a waiting list format. Those in the control group will be offered to participate in the therapy sessions, but only once the intervention and final assessment of the first group have been completed. Those in the intervention group will be divided into groups of 8 and 12 subjects for the group sessions.

### Intervention

The cognitive-behavioral intervention aims at modifying the dysfunctional beliefs in order to appropriately deal with care activities. It is based on the theoretical principles of the cognitive theory, which assumes that specific thoughts and beliefs may trigger negative emotions and unsettled behavior. This program offers the possibility to train cognitive-behavioral capacities and strategies to confront negative feelings and emotions (guilt, sadness, anger) and to change or eliminate certain negative beliefs and thoughts that serve as barriers or obstacles that hinder appropriate care [13,14].

#### Main sessions and contents of the cognitive-behavioral program

Session 1.-Presentation of the program: Introducing the Stress Model

Session 2. Necessity of self-care and introducing the concepts of thought, emotion and situation. Taking into account the necessity of caring, the relation among thought, emotion and conduct, and the differences among these concepts.

Session 3. Learning the cognitive model and differentiating among thought, emotion and situation. Distinguishing the concepts of situation, thought and emotion, the concept of automatic thoughts. Influence of one's body and body states on one's feelings

Session 4. Analyzing the errors of thought. Introducing the importance of pleasant activities.

Session 5.-Learning to adapt our thoughts to reality and to plan pleasant activities. Review of the previous session (errors of thought). Relation between daily activities and state of mind.

Session 6. Intervention in cognitive barriers: "you should". Feelings of guilt and the role of society on these cognitive barriers (errors of thought): What does the "you should" phrase mean and why it is important. Identifying the "you should" phrases, different forms they may take, where they come from and how they have been formed.

Session 7. Knowing our rights and learning to ask for help: personal "you should" phrases, implementing the "you should" phrase, analyzing and discussing the caregivers' rights and the difficulties in applying them into their lives; difficulties in asking for help.

Session 8.-Knowing our rights and learning to ask for help (II): How to ask for help and review.

A coordination system will also be established between the Health Care Center professionals (cotherapists) and the psychologists of the Mental Health Units (therapists) in order to provide caregivers with the necessary help for the specific interventions of the project.

### Measurements

#### 1.Sociodemographic variables

The initial assessment protocol of the caregivers comprises the following sociodemographic variables associated with care: age and sex of the caregivers and the person under care; relationship or kinship with the person under care; number of months and daily hours dedicated to the tasks related with care; cause of the dependence; family members the person under care lives with; help received from relatives, friends or institutions; and characteristics of the caregiver (educational level, professional activity, marital status, consumption of antidepressant or anti-anxiety agents, state of health, income). The type of disease that causes the dependence is also assessed, the dependence degree of the patient through the Barthel Index (which evaluates the dependence for the basic activities of the daily life) and the Pfeiffer Test (to assess the cognitive deterioration).

#### 2. Result Variables

Variables will be assessed before the intervention and after six months of the first assessment. Questionnaires in Spanish will be used as determined by the validations performed in the Primary Health Care System. The following questionnaires are to be used:

a) Family functionality perceived by the caregiver was evaluated with the Family APGAR Questionnaire validated in Spain [15]. This questionnaire rates satisfaction with family relations and distinguish five components of the family function: adaptability, partnership, growth, affection and resolve. It consists of the five questions, with three possible answers: 0 ("hardly ever"), 1 ("sometimes"), 2 ("always"). The total score range varies from 0 to 10, meaning the higher total score, the better family functioning. A global score of 7 points or more indicates family functionality, while a score of less than 7 points indicates family dysfunction. The internal consistency (Cronbach's alpha) of this questionnaire in this study was 0.77.

b)The Short Zarit Interview used in palliative care cases to determine the family giving up has an S of 100%, Sp 90.5%, PPV 95.45%, and NPV 100% in defining caregivers' burden in primary care [16].

c) Quality of life was evaluated with Ruiz and Baca's Questionnaire (1993) [17]. This is made up of 39 items each with a Likert-type five-point scale comprising four dimensions: social support, general satisfaction, physical/mental well-being, work overload and free time. This study gave an internal consistency index (Cronbach's alpha) of 0.94.

d) Social support: in order to measure caregivers' perception of the amount and type of personal social support they receive, the 11-item version [18] of the Duke-UNC Functional Social Support Questionnaire [19] was used. Response options to the items (for example, ''I get love and affection'') are on a 5-point scale ranging from 1 (''much less than I would like'') to 5 (''as much as I would like''). The scale had good internal consistency (Cronbach's a = 0.82).

e) Mental health: caregiver's mental health was measured with the 12-item version of the General Health Questionnaire (GHQ-12) [20], one of the most extensively used screening instruments for common mental disorders. Items (for example, ''being able toconcentrate on whatever you are doing'') are rated by caregivers on a 4-point Likert-type scale, ranging from 0 (''better than usual'', ''not at all'' or ''more than usual'') to 3 (''much less than usual'', ''much more than usual'' or ''much less than usual''). The scale hadgood internal consistency (Cronbach's a = 0.83).

f) Dysfunctional thoughts about caregiving: the dysfunctional thoughts about caregiving questionnaire (DTCQ) [13,14] is a 16-item measure developed following cognitive behavioral principles that assesses caregivers' thoughts that may act as barriers or obstacles to an adaptive coping style with regard to caregiving (e.g., 'A good caregiver should never get mad or lose control with the person who is being cared for'). Responses are coded on a Likert scale that ranges from 0 ('totally disagree') to 4 ('totally agree'). In this questionnaire's development study, this scale showed a 3-month test-retest reliability of 0.60 and a correlation of 0.59 with a brief version of the Dysfunctional Attitudes Scale developed by Andrews, Lewinsohn, Hops, and Roberts (1993). Good internal consistency was found for this scale in this study (Cronbach's alpha 0.90).

#### 3. Implementation measures of the intervention

A variety of assessment procedures has been designed of the achievements accomplished with the program, during the sessions and during the sessions intervals. These procedures aim at responding to three key questions associated with each of the aspects of the intervention implementation defined in the works by Zarit y Leitsch (2001) [21]:

##### 3.1.-Did you understand the concepts mentioned in the program? (Transmission of information)

As part of the satisfaction questionnaire of the program, to be completed anonymously and to be delivered during the last session of the program, caregivers are required to inform how satisfied they are with the content of the sessions, if these were clearly presented, if the number of sessions is sufficient, etc.

##### 3.2.- Did you learn the capacities and techniques taught during the sessions? (Reception of intervention)

The assessment of how the information is received will be carried out by registering the attendance to each session, as well as with a questionnaire specifically designed for this program. The questionnaire serves to assess if after intervention caregivers believe that their knowledge or capacities have increased so as to understand or confront the key aspects the program focuses on.

##### 3.3. - Were techniques and capacities appropriately used in the specific care context? (Generalization of intervention)

Two procedures have been designed to evaluate the completion of the registries the caregivers are required to do as "homework". The first procedure exclusively assesses whether registries have been performed according to the criteria established to this end. The second procedure quantitatively assesses the completion of the main registry provided to the caregiver as "homework", in compliance with the mentioned criteria. This is the most important task to the fulfillment of the objectives included in the program. Moreover, it is the sole task required in all sessions from the beginning of the program to its completion. It refers to the registries related to the differences among situation, thought and emotion, which lead to the 5-column technique (modification of thought).

#### 4. Qualitative information

Besides the aforementioned assessment procedures, both the main therapist and the cotherapist will gather qualitative information relevant to the program objectives and likely to contribute data about the results obtained during the intervention, data which was not accurately registered with the other procedures. The collection of the additional information has been recommended by authors such as Zarit and Leitsch (2001) [21].

### Organization of data collection

The data registered by the researcher will be reviewed by the study instructor so as to detect lacking information or inconsistencies in the data. Lacking information or inconsistencies in the variables included in the protocol will be recovered when possible by the study instructor. The instructor can only alter the original information reported by the researchers once the correction signed by the corresponding researcher becomes available. Once the information has been recovered, the database will be validated in order to assure its quality. Lastly, the data analysis will start. The database is the property of the Research Unit of La Alamedilla.

### Statistical analysis

Data input will be made using the Teleform system (Autonomy Cardiff Vista, California, USA), with a questionnaire previously designed for the project, and exporting the data to the SPSS version 15.0 statistical package (SPSS Inc., Chicago, Illinois, USA) for posterior analysis.

A first descriptive analysis of the socio-demographic characteristics of the study groups will be made. The data will be presented with the mean and standard deviation in the case of quantitative variables, and as frequency distributions for qualitative variables. The Pearson chi-squared test will be used to analyze associations between qualitative variables. The Student t-test for independent samples will be used to compare the means for the two groups. In turn, the analysis of repeated measures will be based on the McNemar test for qualitative data and the Student t-test for paired data in application to quantitative data.

In order to describe the characteristics and intensity of the caregivers' analyzed variables, the sum of the grades as well as its mean will be used, so as to compare the range of grades and make their interpretation easier. Analyzing the impact of the intervention will be carried out through intent to treat analysis.

The contrasting hypothesis will establish an alpha risk factor of 0.05 as the limit of statistical significance.

### Ethical and legal issues

In order to guarantee data confidentiality, all the electronic and paper copies of the protocol, signed informed consent documents and results of the tests made in each of the patients will be kept locked in a safe place, and only the study investigators will have access to the data on the subjects who agree to participate in the study.

The protocol was approved (April 25, 2008) by the Research ethics committee from University hospital of Salamanca, Spain and complies with Spanish data protection law 15/1999 and its recently developed specifications (Royal Decree (RD) 1720/2007). Knowledge and agreement to cooperate has been established with the implicated services, signed by the legal representative of the centre.

### Limitations

Although the simple size of the groups to be studied is quite large, taking into account the studies carried out so far in this type of interventions, it might be insufficient to accomplish definitive results. Due to the studied caregivers' greater accessibility to the health care center, the number of lost cases at the study completion is expected to be low, obtaining this way a somewhat larger simple than that of the studies performed in another context.

The intervention group and the control group will be both examined by the same assessors, who will not be responsible for the sessions. This should render an appropriate internal validity of the study.

The questionnaires used to know what the caregivers' situation is have proved not to be sensitive enough with regards to the changes affecting the caregivers. Changes might occur although caregivers are often satisfied with participating on the therapies. They nevertheless do not match the results obtained.

The greater limitation derives from the increasing tendency in specialized theoretical works indicating that the fact of not obtaining positive results with the interventions might be due to the fact that effectiveness is not assessed on a long-termed objective. The study will perform the final assessment six months after the first assessment.

## Discussion

Publications relating to interventions designed to improve the caregivers' mental health are scarce. Moreover, most of them focus on caregivers of patients suffering from dementia. Some of the publications are characterized by important methodological limitations to establish comparisons among different interventions [1-3,22]. It is important to describe which content and procedures are used in an intervention program and how they are implemented. Taking into account the premise that not everything is equally valid, it is necessary to consider certain variables (such as the time dedicated to the learning of each capacity and strategy used) to measure the effectiveness, which should be performed considering the content and objectives used in the intervention [23,24].

It has been demonstrated that all psychotherapeutic interventions are not effective when reducing the caregiver's discomfort. Psychotherapeutic interventions obtain best results in reducing the symptomatolgy and improving the emotional state of the caregivers. Individual therapies offer better results than group therapies [7].

The significance of our study comes from the fact that it will apply on caregivers of patients familiar with dementia a project proven to be effective in a different context than that of the Primary Health Care System and developed by mental health professionals. If it proves to be effective both with caregivers of patients with and without dementia and with Primary Health Care professionals, it would be an important instrument to be offered to dependent patients.

The content of the intervention sessions, the documents to be completed by caregivers, and the test to be assessed will be applied equally by two professionals in both health care centers. The aim is to assess the reproducibility of such material and determine if the intervention is effective, the possibility to reproduce it in different primary health care centers. If so, it could be subsequently be introduced in general care programs.

Finally, if the results of the intervention are positive, it will be important to know if they persist in time. This would enable the establishment among other things of the financial resources that should be destined to these therapies and compared with other alternatives offered to the relatives who take care of the dependent patients.

## Competing interests

The authors declare that they have no competing interests.

## Authors' contributions

Conception of the idea for the study: ERS and LGO. Development of the protocol, organization and funding: ERS, SMS, NPS, MCP, JIR, CBM, DPA, MAG and LGO. Writing of the manuscript: ERS and LGO All the authors have read the draft critically, to make contributions, and have approved the final text.

## Pre-publication history

The pre-publication history for this paper can be accessed here:

http://www.biomedcentral.com/1471-2458/10/559/prepub
